# Supplementary material for: Newly Developed Restorer Lines of Sorghum [Sorghum bicolor (L.) Moench] Resistant to Greenbug
Source: Plants (Basel). 2024 Jan 31;13(3):425. doi: 10.3390/plants13030425 (PMC10857335; doi:10.3390/plants13030425)
Supplement: Supplementary file 1 [file plants-13-00425-s001.zip › plants-2818018-supplementary.pdf]

Table S1. Chromosome-specific primers used in the study.

Marker type	Name	Chr	Gene	Sequence (5′ - 3′)	Fragment size in parental lines (female/male), bp	Reference
SSR	Xtxp18	8	*Rf1*	F:actgtctagaacaacctgcg<br>R:ttgctctagctaggcatttc	235/237	[5,6]
SSR	Xtxp50	2	*Rf2*	F:tgatgttgttacccttctgg<br>R:agcctatgtatgtgttcgtcc	304/317	[7]
SSR	Xnhsbm 1089	5	*Rf5*	F:catttcacattcaaggtcatgg<br>R:acatttatgggtgcgtgctt	227/224	[8]
SSR	SB2386	4	*Rf6*	F:ggcggtaggtgtaaaaaggaagga<br>R:gcatgccctacgactcttgtgtct	172/166	[9]
SSR	Sb5_236	3		F:gccaagagaaacacaaacaa<br>R:agcaatgtatttaggcaacaca	175/177	[35]
SSR	*Sb6_342*	7		F:tgcttgtgagagtgcctccct<br>R:gtgaacctgctgctttagtcgatg	290/285	[35]
SSR	*Sb6_34*	8		F:aacagcagtaatgccacac<br>R:tgacttggtagagaacttgtcttc	204/194	[35]
STS	2459403	2		F:caggggccaaatgttgttac<br>R: cacagttttatattttccgtgatagtg	935	[26]
CAPS/ MseI	CAPS-572	2		F:caggggccaaatgttgttac<br>R:cacagttttatattttccgtgatagtg	+572/-572	This study
CAPS/ HpaI	CAPS-935	2		F:caggggccaaatgttgttac<br>R:cacagttttatattttccgtgatagtg	935/935+753+182	This study

Table S2. CAPS and SSR polymorphic markers used for Principal component analysis.

№	Sample	Marker/chromosome																	
		CAPS-572<br><br>Chr2		CAPS-935<br><br>Chr2		SSR Xtxp18<br><br>Chr8		SSR-Xtxp50<br><br>Chr2		SSR-Xnhsbm1089<br><br>Chr5		SSR-SB2387<br><br>Chr4		SSR-Sb5_236<br><br>Chr3		SSR-Sb6_342<br><br>Chr7		SSR-Sb6_34<br><br>Chr8	
		allele		allele		allele		allele		allele		allele		allel		allele		allele	
		-572	+572	935+753+182	935	235	237	304	317	227	224	172	166	175	177	290	285	204	194
1	Nizkorosloe 81s	1	0	1	0	1	0	1	0	1	0	1	0	1	0	1	0	1	0
2	929-3	0	1	0	1	0	1	0	1	0	1	0	1	0	1	0	1	0	1
3	928-1	0	1	0	1	0	1	0	1	0	1	0	1	0	1	0	1	0	1
4	R-929-1	0	1	0	1	1	0	0	1	1	0	1	0	1	0	1	0	1	0
5	R-929-2	0	1	0	1	1	0	0	1	1	0	1	0	1	0	1	0	1	0
6	R-929-3	0	1	0	1	1	0	0	1	1	0	1	0	0	1	1	0	1	0
7	R-928-1	0	1	0	1	1	0	0	1	1	0	1	0	1	0	1	0	1	0
8	R-928-2	0	1	0	1	1	0	0	1	1	0	1	0	1	0	1	0	1	0
9	R-928-3	0	1	0	1	1	0	0	1	0	1	1	0	1	0	1	0	0	1
10	R-928-4	0	1	0	1	1	0	0	1	0	1	1	0	1	0	1	0	0	1
11	R-928-5	0	1	0	1	1	0	0	1	0	1	1	0	1	0	1	0	0	1

Table S3. Sequence polymorphisms within the *Rf2* candidate gene fragment

GenBank accession, genotype	Nucleotide position according to GenBank accession number Sobic.002G057050	Reference
	1087-1097*	
Sobic.002G057050	GTT**AA**CCTTAA	https://bioinformatics.psb.ugent.be/plaza/versions/
XM_002459403	GTT**AA**CCTTAA	https://www.ncbi.nlm.nih.gov/
FambeA, CMS line	GTT**AT**TTTTGC	[11]
Lata (R male inbred line)	GTT**TT**TTTTGC	[11]
Nizkorosloe 81s	GTT**AA**CCTTAA	[15]
928-2, R-line	GTT**TT**TTTTGC	[15]
929-3, R-line	GTT**TT**TTTTGC	[15]

* positions 1090 and 1091 are highlighted in bold and underlined

Table S4. Sedimentation values for studied sorghum lines.

Flour	Sedimentation value/Acetic acid variant (AA), ml	Sedimentation value/SDS variant, ml	SDS/AA
Nizkorosloe 81s	43,0±1,0	29,5±0,5	0,7±0,0
929-3	47,0±1,0	46,0±1,0	1.0±0,0
928-1	46,5±1,5	46,5±2,5	1,0±0,0
R-929-1	38,0±0,0	51,0±1,0	1,3±0,0
R-929-2	38,5±2,5	49,0±1,0	1,3±0,1
R-929-3	40,5±0,5	55,5±2,5	1,4±0.1
R-928-1	38,5±0,5	44,5±0,5	1,2±0,0
R-928-2	39,0±1,0	39,0±1,0	1,0±0,0
R-928-3	38,0±2,0	46,5±0,5	1,2±0,1
R-928-4	38,0±0,0	48,0±2,0	1,3±0,1
R-928-5	38,0±0,0	46,0±0,0	1,2±0,0

Table S5. The rheological characteristics of sorghum-wheat flour composites (average value * for studied lines).

Composite ratio (% Sorghum)	Development time (min).	Stability (min).	Liquefaction of the dough in 10 minutes from the start point of the experiment; BU***	Liquefaction of the dough in 12 minutes from the extreme point on the farinogram (from the moment the dough is ready); BU***	Flour quality indicator (mm is Quality Number)	Valorimeter value; BU***
10%	3,20±0,09	1,08±0,05	66,37±5,1	85,50±5,07	44,75±1,28	48,12±1,4
30%	5,34±1,13	3,926±1,31	17,63±6,16	39,12±6,73	143,87±24,56	64,00±3,7
0% **	3,02	0,87	113,00	143,00	39,00	41,00

*sample average value
**Test: wheat flour
***BU – Brabender units
